# The Study of a Novel Nanoparticle-Enhanced Wormlike Micellar System

**DOI:** 10.1186/s11671-017-2198-2

**Published:** 2017-06-30

**Authors:** Caili Dai, Yue Zhang, Mingwei Gao, Yuyang Li, Wenjiao Lv, Xinke Wang, Yining Wu, Mingwei Zhao

**Affiliations:** School of Petroleum Engineering, State Key Laboratory of Heavy Oil Processing, China University of Petroleum (East China), Qingdao, 266580 Shandong People’s Republic of China

**Keywords:** Silica nanoparticle, Nanoparticles enhanced, Rheological properties, Contour length

## Abstract

In this work, a novel nanoparticle-enhanced wormlike micellar system (NEWMS) was proposed based on the typical wormlike micelles composed of cetyltrimethylammonium bromide (CTAB) and sodium salicylate (NaSal). In order to strengthen the structure of wormlike micelles, silica nanoparticles are used to design the novel nanoparticle-enhanced wormlike micelle. The stability and morphologies of silica nanoparticles were studied by dynamic light scattering (DLS) and transmission electron microscopy (TEM) at first. After the formation of NEWMS, the rheological properties were discussed in detail. The zero-shear viscosity of NEWMS increases with the addition of silica nanoparticles. Dynamic oscillatory measurements show the viscoelastic properties of NEWMS. Through comparison with the original wormlike micelles, the entanglement length and mesh size of NEWMS are nearly unchanged, while the contour length increases with the increase of silica concentration. These phenomena confirm the enhanced influence of silica nanoparticles on wormlike micelles. The formation mechanism of NEWMS, especially the interactions between wormlike micelles and nanoparticles, is proposed. This work can deepen the understanding of the novel NEWMS and widen their applications.

## Background

Recently, self-assembly of surfactants has received important and deserved attentions in many experimental, theoretical, and numerous industrial applications. The surfactants can self-assemble to form aggregates with different microstructures. At a concentration above the critical micelle concentration (cmc), they usually form spherical micelles [1]. With the further increase of concentration, surfactant molecules can form aggregates with different morphologies, such as rodlike micelles, wormlike micelles, vesicles, lamellar phases, and liquid crystals [2]. Among these aggregates with various morphologies, viscoelastic wormlike micelles are significant for their special characteristic and wide applications, such as enhancing oil recovery by fracturing, drag reducer, and skincare products [3–6]. Wormlike micelles are long, threadlike aggregates of surfactants or other amphiphiles. These wormlike micelles can entangle with each other to form network structure, showing viscoelastic behavior [7–9]. Comparing with normal polymer solution with viscoelastic features, wormlike micelles can constantly break, reform within an equilibrium process, and recombine under the external conditions [7, 10–12], such as temperature, hydrophobic additives, and high shear rate. When wormlike micelles exist in high temperature or high shear rate, the structure of wormlike micelles will become unstable. Therefore, how to improve the stability of conventional wormlike micelles is still a great challenge [13].

In order to strengthen the structure of conventional wormlike micelles, some groups have done many useful works. Shashkina et al. have studied rheological properties of wormlike micelles by viscoelastic cationic surfactant erucyl bis(hydroxyethyl)methylammonium chloride (EHAC) with the addition of hydrophobically modified polyacrylamide [14]. They observed the polymer could demonstrate an increase trend in viscosity as compared to pure component. In addition, the wormlike micelle prepared by gemini surfactants has become a hot area of research for several years. For the special structure of gemini surfactant, wormlike micelles formed by gemini surfactants can have better viscoelasticity than conventional wormlike micelles [15, 16]. Pei et al. used anionic gemini surfactants to form wormlike micelles, which have good viscoelasticity [17].

In the recent years, nanoparticles have received a large amount of attentions due to their small sizes, resulting in many interesting nanosize effects. The addition of nanoparticles is very explorative to introduce significant changes in macroscopic properties and phase behaviors [4, 18–20]. More recently, some researchers have studied rheological properties of wormlike micelles with the addition of nanoparticles and proposed the mechanisms of interactions between nanoparticles and wormlike micelles. Nettesheim et al. have researched the viscoelasticity of wormlike micelles composed of cetyltrimethylammonium bromide (CTAB) and sodium nitrate (NaNO_3_) with the aid of silica nanoparticles, following the typical Maxwell fluid model. Both the zero-shear rate viscosity (*η*_0_) and relaxation time (*τ*_R_) of solutions increase after the addition of silica nanoparticles [21]. Helgeson et al. further conducted structural and thermodynamic measurements in CTAB/NaNO_3_ wormlike micellar solution within dilute silica nanoparticles. They found the formation of micelle-nanoparticle junctions acting as physical cross-links between micelles [22], which were observed by cryogenic transmission electron microscopy (cryo-TEM). Luo et al. used barium titanate (BaTiO_3_) nanoparticles to modify wormlike micelles by the anionic surfactant fatty acid methyl ester sulfonate sodium and investigated influence of different factors on the viscoelasticity of wormlike micelles, such as concentration of surfactant, mass fraction of nanoparticles, and temperature. Fan et al. found that silica nanoparticles can induce micellar growth in NaOA (sodium oleate) wormlike micelles solutions, enhancing the bulk viscosity [23]. Pletneva et al. have investigated novel viscoelastic smart suspensions based on cationic wormlike micelles with the addition of oppositely charged submicron magnetic particles [24]. Fei et al. investigated the potential of silica nanoparticles to stabilize foam under high-temperature conditions. They found that the SiO_2_ nanoparticles and wormlike micelles exhibit a synergistic effect in terms of foam rheology and stability, which significantly improves the proppant suspension capabilities for petroleum applications [25]. However, there have been not many researches on the effects of silica nanoparticles on wormlike micelles at different concentrations so far.

In this work, the novel nanoparticle-enhanced wormlike micellar system (NEWMS) was studied. The conventional wormlike micelle is formed by CTAB and sodium salicylate (NaSal), which is one of the most widely applied formulas at present [26, 27]. NEWMS were prepared by 50 mM CTAB and 60 mM NaSal with the addition of silica nanoparticles. Dynamic light scattering (DLS) and transmission electron microscopy (TEM) were used to study the silica nanofluids. Rheological measurements were conducted to evaluate the rheological properties of NEWMS. The effects of different silica concentrations on the entanglement length, mesh size, and contour length of wormlike micelles are clarified.

## Methods

### Material

CTAB and NaSal were purchased from Shanghai Experimental Reagent Co., Ltd., without further purification. Silica nanoparticles with a diameter of 7–40 nm were supplied by Aladdin Industrial Co., Ltd. Water was triply distilled.

### Sample Preparation

Silica nanofluids are prepared by simply dispersing silica nanoparticles in water at different mass fractions, including 0.1, 0.3, and 0.5%. After mixing by mechanical stirrer at 340 rpm for 30 min and dispersing by ultrasonic dispersion for 3 h, transparent silica nanofluids are prepared. NEWMS are prepared according to the following steps: silica nanofluid is regarded as the base fluid, which is used to prepare CTAB solution (100 mM) and NaSal solution (120 mM). After adding CTAB or NaSal into silica nanofluid, the solution is dispersed by ultrasonic dispersion for 10 min at 35 °C. Then, CTAB solution and NaSal solution are mixed in equal volume. After mixing for 30 min, NEWMS were prepared. In addition, wormlike micelle of CTAB and NaSal without silica nanoparticles was regarded as a contrast sample.

### Characterizations

#### Transmission Electron Microscopy

The transmission electron microscopy (TEM) image of silica nanoparticles was characterized using a JEOL microscope (JEM-2100).

#### Dynamic Light Scattering Measurements

DLS measurements were performed on the Zetasizer Nano ZS (Malvern, UK) with a laser light wavelength of 633 nm and a scattering angle of 90°. The sample was transferred to a square sample pool, and the measurement was repeated three times. All measurements were conducted at 25 ± 0.1 °C.

#### Rheological Measurements

The rheological properties of samples were measured by using Haake Mars 60 rheometer with the cone plate system (diameter, 35 mm; angle, 1°). The temperature is kept at 25 ± 0.05 °C with Peltier-based temperature controlling. The range of shear rate is kept from 0.01 to 100 s^−1^ during the steady shear measurement. In oscillatory measurements, the frequency was kept at 6.28 rad s^−1^ (1 Hz) with the variation of stress (*σ*). When the linear viscoelastic region was confirmed, frequency sweep measurements were performed as a function of frequency at a constant stress. In addition, before rheological measurements, what calls for attention is that all wormlike micelles solutions in this work should be put in the thermostat at 25 °C for 24 h, ensuring the formation of micelles and stability of micelle-particle junctions.

## Results and Discussion

### Formation of Silica Nanofluids

**Fig. 1:**
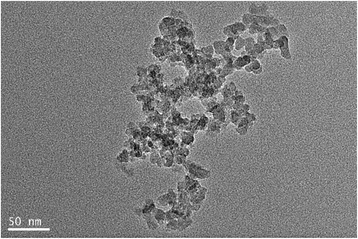
The TEM micrograph of silica nanoparticles

At first, silica nanofluids were characterized by TEM and DLS. The TEM image of silica nanoparticles is shown in Fig. 1. It can be observed that most of nanoparticles suspending in the solution have a uniform size. Because of the strong interactions among nanoparticles, larger silica aggregates are developed [4, 28–30]. Table 1 lists the average size of silica nanoparticles and polydispersity index (PDI) at different silica concentrations. It is clear that the average size of silica nanoparticle solution becomes bigger gradually with the increase of concentration, which reflects the different aggregation levels of silica nanoparticles.

**Table 1:** The average size, polydispersity index (PDI), and zeta potential of solutions with different silica concentrations at 25 °∁

C_silica_ (wt%)	0.1	0.3	0.5
Average size of nanoparticles (nm)	127.34	170.38	192.33
Polydispersity index	0.117	0.13	0.165
Zeta potential (mV)	−20.15	−24.45	−20.85

The zeta potentials of solutions are listed in Table 1. According to references, the electrostatic repulsive interactions between nanoparticles can keep particles from frequent collisions, aggregation, and sedimentation [4, 31]. The zeta potential is the potential difference between the dispersion medium and the stationary layer of fluid attached to the dispersed particle, which is associated with the stability of colloidal dispersion [32–34]. The larger the absolute value of zeta potential is, the more stable the solution is. As shown, the zeta potential of nanofluids at 0.3 wt% is higher than that of other two samples, indicating that 0.3 wt% silica nanofluid is more stable.

### Properties of NEWMS

**Fig. 2:**
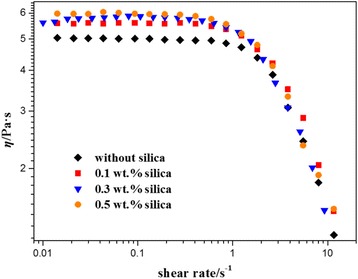
Steady shear viscosities of wormlike micelle solutions with addition of different silica mass fraction at 25 °∁

In order to study the influence of silica nanoparticles on NEWMS, steady shear measurements of fluids are conducted firstly. Viscosities of NEWMS with different shear rates are shown in Fig. 2. At low shear rates, viscosities can keep constant. This plateau value of viscosity is generally regarded as zero-shear viscosity (*η*_0_). With the increase of shear rate, viscosities become smaller and show remarkable shear-thinning phenomenon, which is the typical symbol of wormlike micelle formation [7, 35–39]. While at high shear rates, the reduction of viscosities can be due to the alignment of wormlike micelles, which results in the shear-banding phenomenon [18, 40–42]. Through comparison, at low shear rates, the plateau value of viscosities become larger with the increase of silica nanoparticle concentration. It indicates that the viscosity of NEWMS varies extremely depending on the silica concentration.

**Fig. 3:**
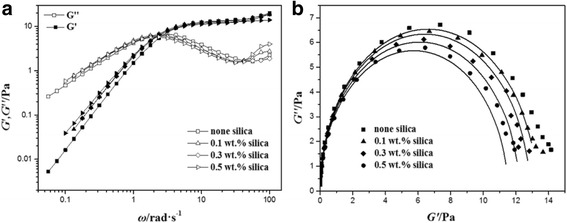
Variations of *G*′ (filled symbols) and *G*″ (open symbols) with shear frequency and Cole–Cole plots for NEWMS with different silica concentrations at 25 °∁

In order to investigate viscoelastic properties, dynamic rheological oscillatory measurements were conducted. As shown in Fig. 3a, storage modulus *G*′ and loss modulus *G*″ vary with oscillation frequency and all NEWMS exhibit typical features of wormlike micelles. At low frequencies, *G*″ is much larger than *G*′, which shows that wormlike micelles have more viscous properties [43–47]. While at high shear rates, *G*′ is larger than *G*″, showing more elastic properties. With the increase of silica concentrations, the values of *G*′ and *G*″ become larger slightly under the same shear frequency, illustrating that the addition of silica nanoparticles affects the viscoelasticity of wormlike micelles. Until at larger frequencies, *G*′ reaches a plateau modulus *G*_0_. Meanwhile, *G*″ reaches a minimum value, determined as *G*″_min_.

For wormlike micelles, a typical Maxwell model is generally used to study rheological properties. The moduli *G*′ and *G*″ can be calculated according to the following Eqs. 1 and 2 [48]:

1 $$ G^{\prime }=\frac{G_0{\omega}^2{\tau}_{\mathrm{R}}^2}{1+{\omega}^2{\tau}_{\mathrm{R}}^2} $$

2 $$ G^{{\prime\prime} }=\frac{G_0\omega {\tau}_{\mathrm{R}}}{1+{\omega}^2{\tau}_{\mathrm{R}}^2} $$

The Cole–Cole plot is usually used to study whether *G*′ and *G*″ fit the Maxwell model well. The Cole–Cole plot (the curve of *G*″ as a function of *G*′) is studied from the following Eq. 3 [48]:

3 $$ G^{{\prime\prime} }+{\left( G\prime -\frac{G_0}{2}\right)}^2={\left(\frac{G_0}{2}\right)}^2 $$

Figure 3b shows the plots of *G*″ versus *G*′ of NEWMS with different silica concentrations, where experimental results are shown in points and solid lines are calculated and fitted according to Eq. 3. At low frequencies, the experimental plots fit well with the calculated Cole–Cole plots, following the Maxwell model well. However, at high shear frequencies, experimental data deviate from semicircle in the Cole–Cole plots. This phenomenon may be attributed to the Rouse relaxation modes or “breathe modes” [41, 49].

For the Maxwellian linear viscoelastic micelle, the breakage time *τ*_break_ is much less than the reputation time *τ*_rep_ *. τ*_break_ can be calculated from equation *τ*_break_ *= ω* ^−1^, where the frequency *ω* corresponds to *G*″_min_. As shown in Eq. 4, these parameters are also associated with the single relaxation time *τ*_R_.

The relaxation time *τ*_R_ is an important rheological parameter for evaluating properties of wormlike micelles, which can be calculated according to the following Eq. 5 proposed by Cates [1]:

4 $$ {\tau}_{\mathrm{R}}=\sqrt{\tau_{\mathrm{rep}}{\tau}_{\mathrm{break}}} $$

5 $$ {\tau}_{\mathrm{R}}=\frac{\eta_0}{G_{\infty}^{\prime }} $$

*G*′_*∞*_ can be calculated from the equation *G*′_∞_ = 2 *G*″_max_, in which *G*″_max_ is the modulus while *G*′ is equal to *G*″. In addition, the mesh size *ξ*_M_, the entanglement length *l*_e_, the persistence length *l*_p_, and the contour length *L* are important parameters for measuring the wormlike micelles in NEWMS. Rubber elasticity relates the mesh size *ξ*_M_ directly to the plateau modulus and the network density *ν* as [1, 48]

6 $$ {G}_{\infty}^{\prime }= v{k}_B T\propto \frac{k_B T}{\xi_{\mathrm{M}}^3} $$

The value of *k*_*B*_ is 1.38 × 10^−23^ J/K as the Boltzman constant. *T* is the absolute temperature, the value of which is 298 K in this work. The loss modulus at the minimum is related to the contour length *L* and entanglement length *l*_e_, which is shown as Eq. 7. The entanglement length is related to the mesh size *ξ*_M_ and persistence length *l*_p_ by Eq. 8 [48, 50].

7 $$ \frac{G_{\infty}^{\prime }}{G_{\min}^{{\prime\prime} }}\approx \frac{L}{l_{\mathrm{e}}} $$

8 $$ {l}_e=\frac{\xi_M^{5/3}}{l_p^{2/3}} $$

Here, *l*_p_ is set 15–25 nm according to previous references [44]. Above all, calculations of these parameters are listed in Table 2.

**Table 2:** The rheological parameters of wormlike micelles with different silica concentrations

C_silica_ (wt%)	0	0.1	0.3	0.5
*η*_0_ (Pa s)	5.011	5.598	5.787	5.978
*G*_0_ (Pa)	13.31	13.01	12.66	12.57
*G*′_∞_ (Pa)	13.11	13.02	12.51	12.53
*G*″_min_ (Pa)	1.651	1.569	1.529	1.504
*τ*_R_ (s)	0.378	0.436	0.475	0.521
*τ*_rep_ (s)	4.609	6.016	7.140	8.590
*τ*_break_ (s)	0.031	0.032	0.032	0.032
*l*_e_ (nm)	132–185	134–188	138–194	143–201
*ξ*_M_ (nm)	67.9	68.1	69.0	68.9
*L* (nm)	1048–1469	1102–1550	1129–1587	1191–1675

**Fig. 4:**
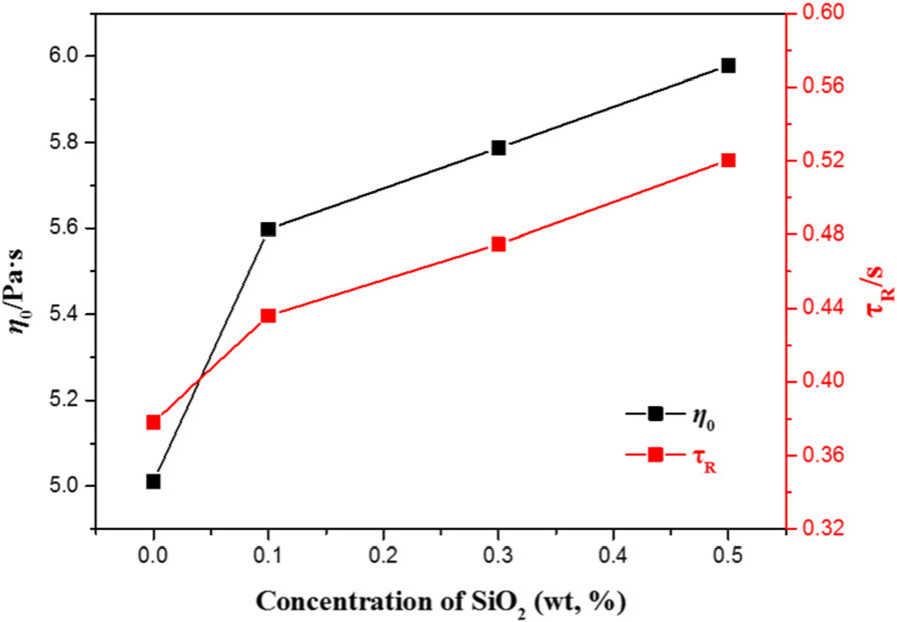
Dependences of the zero-shear viscosity *η*_0_ and the relaxation time *τ*_R_ on the concentration of silica nanoparticles at 25 °∁

As shown in Table 2, adding different mass fractions of nanoparticles does not change plateau modulus significantly. A slight increase of the relaxation time *τ*_R_ gradually is observed with the addition of silica nanoparticles. The measurement of *τ*_break_ shows no significant change. According to Eq. 1, the observed increase in *τ*_R_ with the addition of nanoparticles is primarily due to the increase in *τ*_rep_. As shown in Fig. 4, the addition of silica nanoparticles indeed affects the properties of NEWMS, which reflected in the relaxation time *τ*_R_ and zero-shear viscosity *η*_0_. Through calculation, the values of the parameters *l*_e_ and *ξ*_M_ do not show large changes by adding nanoparticles. While the contour length *L* shows an increasing trend with the increase of silica concentration. This may be the reason why *τ*_R_ increases after adding silica nanoparticles.

### Mechanism Discussion

According to previous studies, the mechanism of viscosity increase with addition of nanoparticles has not been identified yet. Bandyopadhyay and Sood proposed that the increase of viscosity resulted from additional electrostatic screening through contributions of silica nanoparticles to the bulk ion concentration [51]. Helgeson et al. proposed that the addition of nanoparticles not only changed the surface electrical behavior of micellar molecules but also formed a new kind of physical cross-link micellar structure, which could be also called “double network” [22].

**Fig. 5:**
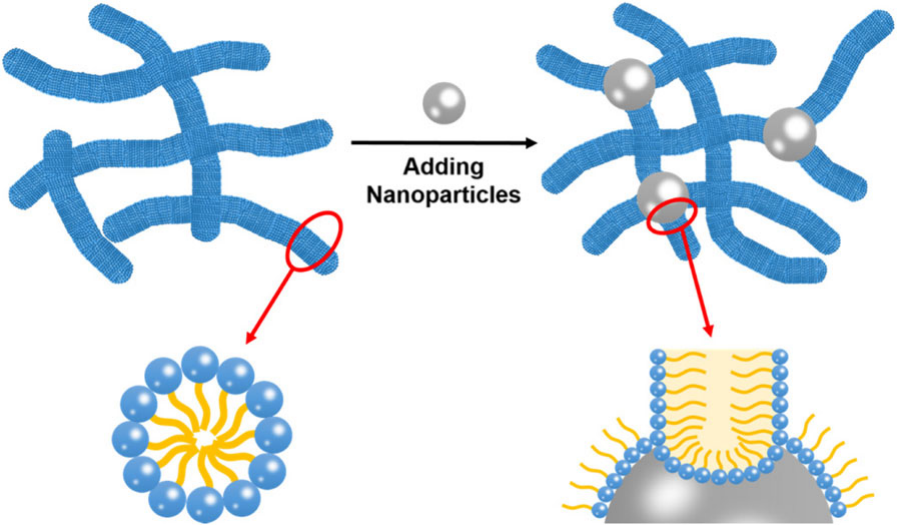
Illustration of the proposed mechanism of a complex cross-linking networks constructed by wormlike micelles and silica nanoparticles

In this work, the improvement of micellar viscoelasticity is noticeable, which reflected in the increase of *η*_0_, *τ*_R_, and *L*. Considering hydrophilic interactions between headgroups and hydrophilic silica nanoparticles, the endcap of wormlike micelle can absorb on the surface of nanoparticles. As shown in Fig. 5, wormlike micelles can grow linear with the addition of surfactant because of the unfavorable energy of formation of endcaps relative to cylinders. When adding silica nanoparticles, nanoparticles can associate with endcaps of wormlike micelles, forming micelle-particle junctions. These micelle-nanoparticle junctions exist in micelles just like joint points, improving the entanglement due to overlapping micelles. In addition, micelle-nanoparticle junctions can significantly entangle more micelles, creating extra viscoelasticity. It is considered that particles with junctions can join the structure between two micelles, causing more efficiently longer micelles. With the increase of silica concentration, it can be considered that the number of micelle-nanoparticle junctions would get increased, further improving the viscosity of NEWMS. In addition, the adsorption of hemispherical endcaps of micelles on the surface of silica nanoparticles may change the electric properties between the micelles, resulting in increased micellar entanglement.

## Conclusions

In conclusion, a novel NEWMS by 50 mM CTAB and 60 mM NaSal with the aid of silica nanoparticles was proposed. Rheological properties show that NEWMS have higher viscosity and better viscoelasticity than conventional wormlike micelles without silica nanoparticles. The addition of silica nanoparticle can cause a remarkable change for the zero-shear viscosity and relaxation time. In addition, a slight increase can be observed from the calculation of the contour length of wormlike micelles. The formation of micelle-nanoparticle junctions improves the entanglement of wormlike micelles and creates extra viscoelasticity. This work could develop further the knowledge of mechanism between wormlike micelles and nanoparticles.

## Abbreviations

cmc: Critical micelle concentration
cryo-TEM: Cryogenic transmission electron microscopy
DLS: Dynamic light scattering
NEWMS: Nanoparticle-enhanced wormlike micellar system
TEM: Transmission electron microscopy
